# Life Cycle Assessment of Functionalized Bionanocompounds with Ice Nucleation Protein for Freezing Applications

**DOI:** 10.3390/polym15061457

**Published:** 2023-03-15

**Authors:** Olga P. Fuentes, Johann F. Osma

**Affiliations:** 1 Department of Electrical and Electronic Engineering, Universidad de los Andes, Cra. 1E No. 19a-40, Bogota 111711, Colombia; 2 Department of Biomedical Engineering, Universidad de los Andes, Cra. 1E No. 19a-40, Bogota 111711, Colombia

**Keywords:** life cycle assessment (LCA), bionanocompounds, magnetite nanoparticles, silica nanoparticles, energy saving, operation stage

## Abstract

The objective of this study was to assess the effectiveness of functionalized bionanocompounds with ice nucleation protein (INP) as a novel approach for freezing applications in terms of how much energy is used during each step of freezing when water bionanocompound solutions were compared with pure water. According to the results of the manufacturing analysis, water required 28 times less energy than the silica + INA bionanocompound and 14 times less than the magnetite + INA bionanocompound. These findings showed that water used the least energy during the manufacturing process. In order to determine the associated environmental implications, an analysis of the operating stage was also conducted, taking the defrosting time of each bionanocompound during a 4 h work cycle into account. Our results showed that bionanocompounds may substantially reduce the environmental effects by achieving a 91% reduction in the impact after their use during all four work cycles in the operation stage. Additionally, given the energy and raw materials needed in this process, this improvement was more significant than at the manufacturing stage. The results from both stages indicated that, when compared with water, the magnetite + INA bionanocompound and the silica + INA bionanocompound would save an estimated 7% and 47% of total energy, respectively. The study’s findings also demonstrated the great potential for using bionanocompounds in freezing applications to reduce the effects on the environment and human health.

## 1. Introduction

Nanomaterials are widely used in current research due to their unique features, including their excellent chemical and physical stability, large surface area, and better efficiency than conventional bulk materials [1,2,3,4]. Nanomaterials can occur naturally, but they can also be created as an unintentional by-product of mechanical or industrial processes (incidental), or can be artificially synthesized through engineering (engineered nanomaterials) [5]. Depending on their chemical composition, engineered nanomaterials can be classified into organic-based and inorganic-based nanomaterials. The latter include metal oxide nanoparticles, which have unique chemical, optical, and electrical properties when compared with bulk materials [6]. Several metal oxide nanoparticles, such as ZnO, MnO_2_, Fe_3_O_4_, TiO_2_, Al_2_O_3_, and SiO_2_, have been studied to detect different molecules [7]. Specifically, in this study, magnetite (Fe_3_O_4_) and silicon dioxide or silica (SiO_2_) nanoparticles were used for their surface functionalization. 

Several studies have shown that magnetite nanoparticles exhibit great potential for the development of new materials, mainly due to their magnetic properties and the possibility of functionalizing their surface with different agents such as enzymes [8], biopolymers [9], organic particles [10], chelating moieties [11], and alkoxysilanes [12]. Similarly, silica nanoparticles have drawn significant attention due to their stability, low toxicity, and ability to be functionalized with a range of molecules and polymers, which can form improved biomaterials from various hybrid nanomaterials [13]. These chemical and physical properties of magnetite and silica nanoparticles allow surface functionalization for applications in freezing.

The ice nucleation protein (INP) anchored to the outer cell membrane of *Pseudomonas syringae* has gained scientific interest, due to its ability to induce the formation of ice crystals that are close to their melting point [14]. The *P. syringae* bacterium is an ice nucleation-active (INA^+^) pathogen that is capable of synthesizing secretory INP. This protein enables bacteria to nucleate crystallization in supercooled water. The snowmaking industry uses it to promote ice nucleation for artificial snow and to economize on water. In this study, the commercially available bacterium Snomax is used, which is the freeze-dried form of *P. syringae* that works at −0.6 °C. INP has been widely used in multidisciplinary studies such as biosynthesis, regulation, pathogenicity, and the production of frozen goods and snowmaking [15]. However, despite the ample number of emerging applications for INP, much work is still needed to ensure effective freezing processes that are economically feasible and sustainable in the long run. Therefore, developing new bionanocompounds which comply with sustainability principles and are cost-effective for freezing is an international priority.

Some studies have reported the effects of freezing techniques on the environment, human health, and the use of energy resources that have an impact on CO_2_ emissions in order to determine which is more environmentally friendly [16,17]. Therefore, this study aimed to compare the environmental impacts associated with the freezing process of two bionanocompounds, one using magnetite-based nanoparticles and the other one being silica-based. This assessment was accomplished through a “cradle to gate” approach, which required a detailed inventory of each raw material required for the synthesis and freezing process of each bionanocompound. Additionally, the mass balance of the processes, or the input and output flow of materials, was determined as part of the life cycle inventory. Moreover, the potential environmental impacts were estimated through the life cycle assessment (LCA) methodology based on the ISO 14040 framework [18]. The results of both bionanocompounds’ freezing processes were compared with a conventional freezer, and the environmental implications were determined. Finally, this study expected to propose more robust, efficient, and reliable processes for freezing while considering their environmental implications.

## 2. Materials and Methods

### 2.1. Materials 

Iron (III) chloride hexahydrate (97%) (FeCl_3_·6H_2_O), tetramethylammonium hydroxide (TMAH) (25%), and (3-aminopropyl) triethoxysilane (APTES) (98%) were purchased from Sigma-Aldrich (Burlington, MA, USA). Iron (II) chloride tetrahydrate (98%) (FeCl_2_·4H_2_O), glutaraldehyde (25%) (GLU), and sodium hydroxide (NaOH) (98%) were obtained from PanReac AppliChem (Barcelona, Spain). Commercial silica dioxide nanoparticles (100 nm) were acquired from Guangzhou Hongwu Material Technology Co., Ltd. (Guangzhou, China). The ice nucleation protein was acquired from the Snowmax company. Magnetite nanoparticles were synthetized according to the process explained in the next section. 

### 2.2. Synthesis of Fe_3_O_4_ Nanoparticles and Surface Modification

Magnetite nanoparticles were synthesized by batch chemical co-precipitation following the procedure reported in the study of Sotelo et al. [19]. Briefly, solutions were created by dissolving 20 mL of 2 M FeCl_3_·6H_2_O and 20 mL of 1 M FeCl_2_·4H_2_O in Milli-Q water. Then the solutions were then magnetically stirred; subsequently, 40 mL of 8 M NaOH was dripped into the chloride mixture with a syringe pump (78-8110C Programmable Touch Screen, Cole-Parmer®, Vernon Hills, IL, USA) at an infusing flow rate of 0.2 mL/min for 60 min. This solution was stirred at 1500 rpm and 90 °C on a stirring hot plate. After this time, magnetite nanoparticles were formed, which were washed 30 times with Milli-Q water. The obtained nanoparticles were precipitated with a magnetic field aided by a neodymium magnet attached to the bottom of the container. 

The surface modification procedure was adapted from the patent of Pulido et al. [20]. First, magnetite and silica nanoparticles were sonicated with Milli-Q water and a 2% (*v*/*v*) TMAH solution for 20 min at 30 °C. Then APTES was added, and the solution was sonicated for 10 min at 30 °C. After silanization, GLU 2% (*v*/*v*) was added and mixed with a vortex for 1 min. This solution was left undisturbed for 1 h at 4 °C. Each sample was treated further with INA protein at 10:1 (*w*/*v*) in a vortex for 1 min and left standing overnight at 4 °C. Finally, magnetite + INA and silica + INA bionanocompounds were formed by anchoring the INA protein to the linker and to the crosslinking agent GLU, which was linked to the APTES linker, which was linked to each substrate.

### 2.3. LCA of Bionanocompounds 

#### 2.3.1. Goal and Scope Definition

**Figure 1:**
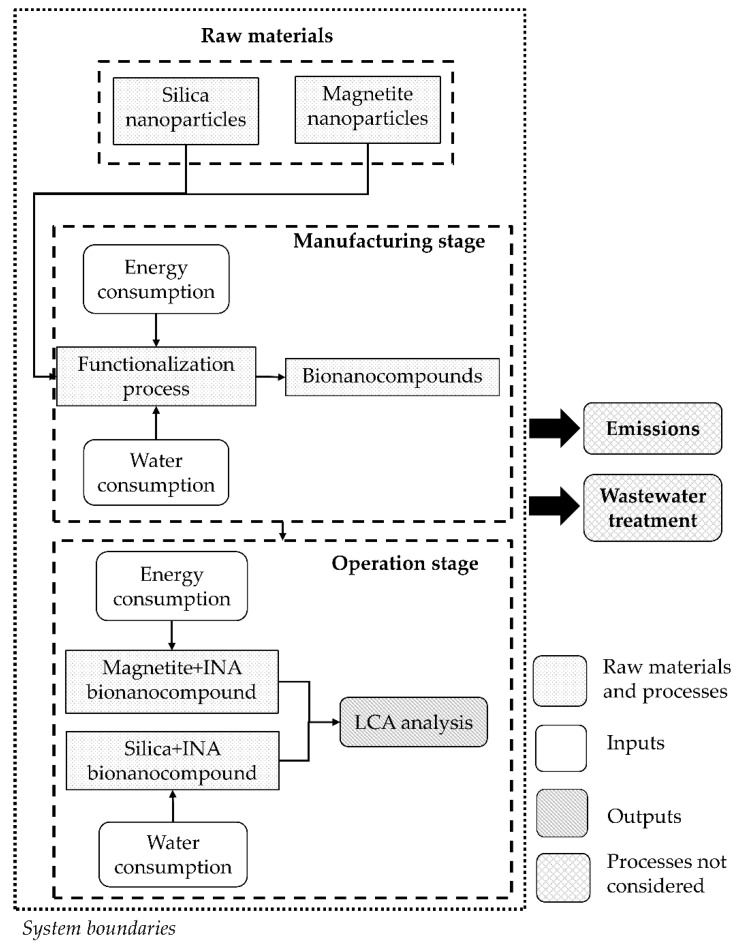
Flow diagram of the manufacturing and operation stages for usage of the bionanocompounds.

This life cycle analysis of bionanocompounds used for freezing applications evaluated the possible environmental implications related to the manufacturing and operation stages. The functional unit for this study was defined as 100 mg of bionanocompounds produced per batch and was based on an attributional approach or descriptive “cradle to gate” analysis of laboratory-scale processes. Additionally, the system’s boundaries were established from the use of raw materials to the operation stage of these bionanocompounds, considering the consumption of water and energy as well as any potential effects on the environment and human health. Figure 1 shows the flow diagram for the specific stages in the synthesis of bionanocompounds and their LCA. According to the defined system boundaries, this study excluded assessments of emissions and wastewater treatment.

#### 2.3.2. Life Cycle Inventory

Data related to the synthesis of bionanocompounds were taken from the patent of Pulido et al. [20], and the quantities of raw materials used to operate the process were considered within the system’s boundaries. Table 1 reports the inventory according to the raw materials, water, and energy consumption of each stage of bionanocompounds used for freezing applications. 

#### 2.3.3. Impact Assessment

The life cycle impact assessment (LCIA) aimed to determine the possible potential impacts on the environment and human health that may result from the manufacturing and use of bionanocompounds used for freezing applications. Background data for the chemicals, electricity, and water were obtained from the Ecoinvent 3.6 database. The selection of the impact categories, category indicators, and characterization models used in this study accurately represented the relevant environmental impact of the system. Therefore, the impact assessment tools were based on the characterization factors provided by the International Reference Life Cycle Data System (ILCD) method for LCIA. Eight impact categories were considered in this study: non-carcinogenic human toxicity effects (HTNc), carcinogenic human toxicity effects (HTCe), ecotoxicity of freshwater (EF), total climate change (CH), resource depletion of minerals and metals (RDMm), resource depletion of dissipated water (RDDw), freshwater and terrestrial acidification (FTA), and photochemical ozone formation (POF).

Regarding the assumptions, data concerning the environmental effects included the production of chemicals required to synthesize the magnetite and silica nanoparticles. However, as information on the environmental effects of producing INP protein has not yet been reported, it was omitted from the LCA analysis.

## 3. Results

### 3.1. Results for Energy Consumption 

**Figure 2:**
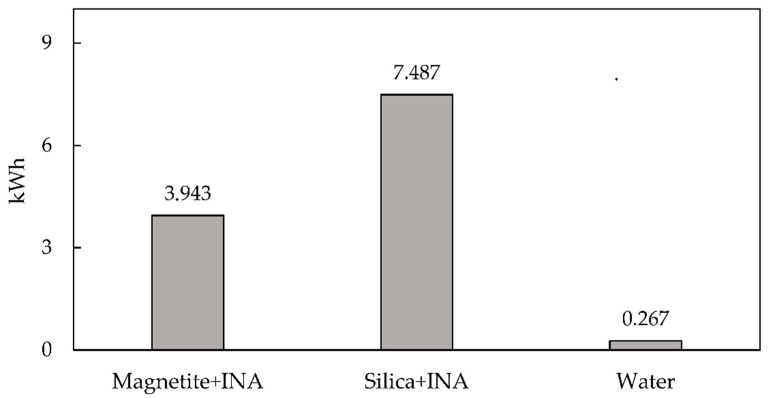
Energy consumption of the manufacturing stage for bionanocompounds and water.

In terms of energy usage, water and bionanocompounds were compared. The defrosting time for ice packs containing 40 mL of each bionanocompound solution and water was calculated for 4 h work cycles. The energy consumption during the manufacturing process is shown in detail in Figure 2. The results showed that the silica + INA bionanocompound consumed around twice as much energy as the magnetite + INA bionanocompound. The energy used by the machinery and the refrigeration stage can be used to explain this outcome. In comparison, the energy consumption of water was 14 times lower than that of the magnetite + INA bionanocompound and 28 times lower than that of the silica + INA bionanocompound. Due to the fact that the analysis only considered the energy used during freezing and not the manufacturing process, these results showed that water had the lowest energy usage when compared with other bionanocompounds.

**Figure 3:**
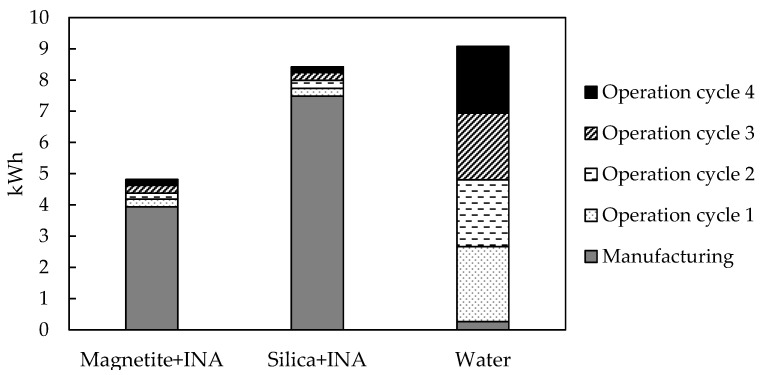
Energy consumption of the operation stage during four work cycles.

According to the experimental tests, the magnetite + INA bionanocompound defrosted in 70 min, the silica + INA bionanocompound defrosted in 65 min, and water defrosted in 30 min. To determine the associated environmental impacts, an analysis of the operation stage was conducted, considering these defrosting times within a work cycle. Figure 3. shows the results for energy consumption during the operation stage in four work cycles. A work cycle represents the time that each bionanocompound and water took to defrost. The results showed that the initial work cycle for the bionanocompounds used 16 times less energy than their manufacturing stage. In contrast, the first work cycle for water required nine times as much energy as used in its manufacturing. These findings are explained by the sort of refrigeration equipment required, as the bionanocompounds needed a conventional refrigerator whereas water required a freezer to freeze. These requirements therefore resulted in higher energy consumption for water during the operation stage than for bionanocompounds.

Figure 3 shows that the manufacturing stage contributed most to the overall effect of bionanocompounds, contributing 82% in the case of the magnetite + INA bionanocompound and 89% in the case of the silica + INA bionanocompound. Water, on the other hand, had a contribution of 3% in the manufacturing stage, since the results only considered the energy used by freezer equipment. Although there were four work cycles for the bionanocompounds, the operation stage had the lowest energy use, according to the results. In particular, the operation stage of the magnetite + INA bionanocompound represented 18%, that of the silica + INA bionanocompound represented 11%, and that of water represented 97%. Overall, bionanocompounds had a 90% lower impact in the operation stage than water. This finding is explained by the prolonged defrosting time of these bionanocompounds during each work cycle.

When the two stages were compared, it was shown that the magnetite + INA bionanocompound and the silica + INA bionanocompound consumed 15 times and 30 times less energy, respectively, during the operation stage than in the manufacturing stage. These findings demonstrated that whereas bionanocompounds consumed more energy during the manufacturing stage, their energy use decreased significantly during the operation stage. Finally, the results suggested that the estimated total energy savings by the magnetite + INA bionanocompound and the silica + INA bionanocompound compared with water were 47% and 7%, respectively.

### 3.2. Results of the Life Cycle Impact Assessment 

**Figure 4:**
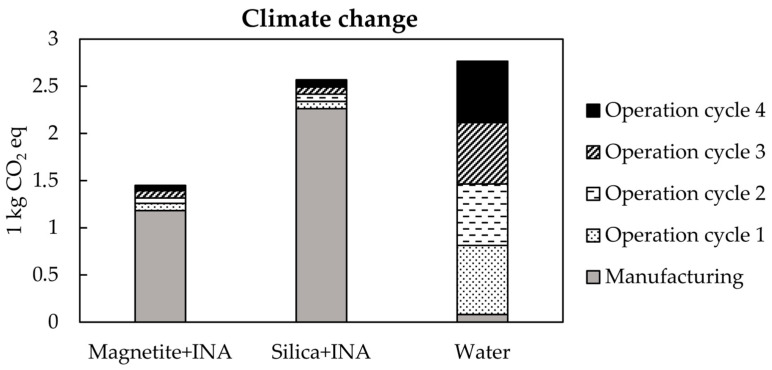
Environmental impacts of the magnetite + INA bionanocompound, the silica + INA bionanocompound, and water on climate change.

Human health, ecosystem quality, climate change, and resource depletion were the four broad categories used for the impact assessment. Figure 4 details the effects of the magnetite + INA bionanocompound, silica + INA bionanocompound, and water on climate change in terms of their manufacturing and operation stages. In general, bionanocompounds contributed significantly more than water in the manufacturing stage. Therefore, the impacts of water in the manufacturing stage were negligible. Regarding the operating stage, water use contributed almost 83% of the total contribution, followed by the silica + INA and magnetite + INA bionanocompounds, which each contributed 9% and 8%, respectively. 

Table 2 and Supplemental Figures S1 and S2 compile the results of the impact assessment for all impact categories in the manufacturing and operation stages of the magnetite + INA bionanocompound, the silica + INA bionanocompound, and water. The results showed that, when compared with the bionanocompounds, water contributed approximately 41% to all categories. This contribution was mainly attributed to the reduced defrosting time per work cycle in the operation stage. These findings highlight the importance of taking the whole lifecycle of a bionanocompound into account, from manufacturing to the final operation.

## 4. Discussion

Different results were found at each stage when comparing the bionanocompounds and water. The results indicated that water consumed 28 times less energy during the manufacturing stage than the silica + INA bionanocompound and 14 times less than the magnetite + INA bionanocompound. These results are explained by the low energy use for freezing water. Therefore, the process that consumed the most energy and consequently had the greatest impacts was the synthesis of bionanocompounds. Similarly, the study of Feijoo et al. [21] demonstrated that over 90% of the life cycle burden associated with producing magnetic nanoparticles were attributable to the environmental effects related to the use of energy and chemicals.

In terms of the results of the assessment of the manufacturing stage impact, the silica + INA bionanocompound contributed 64% to all impact categories. This bionanocompound specifically influenced the human toxicity category 10 times more than the water resource depletion category. Due to the use of silica nanoparticles and their effects on human health, this result can be explained According to several studies, exposure to silica nanoparticles causes a number of disorders, including lung inflammation and heart damage, and has a direct impact on cellular function through DNA damage, metabonomics, oxidative stress, and apoptosis [22,23,24]. The hepato- and nephrotoxic effects of these nanoparticles are caused, in part, by these adverse effects of silica exposure [24]. 

Considering synthesis methods based on environmentally friendly techniques is an alternative to reduce the negative effects that the manufacturing stage of bionanocompounds has on the environment and human health [25,26]. According to recent studies, green synthesis may improve the environmental effects of the production of magnetite nanoparticles [27]. In this synthesis, ecologically friendly components, including stabilizers and reducing agents, were used instead of the conventional raw materials which are frequently used as precursors. Therefore, this alternative can improve the conditions for the production of magnetite, maintaining the original properties and performance while also significantly reducing the environmental effects.

This study addressed an analysis of the operation stage of bionanocompounds, taking several work cycles into account, as proposed in previous works, in order to improve the environmental performance [28]. According to some studies, the usage of ice nucleation proteins (INPs) is a potential strategy to increase the effectiveness of the freeze-drying process by producing an estimated overall energy savings of 28.5% [29]. However, our results showed that bionanocompounds required 90% less energy than water during the operation stage. Therefore, this study indicated a significant decrease in the energy consumption of the use of bionanocompounds. This reduction was attributed to the high defrosting time of bionanocompounds, which led to silica + INA and magnetite + INA using 91% and 92% less energy, respectively, after 16 h of operation.

## 5. Conclusions

This study provides important details on the LCA of bionanocompounds used in freezing applications. Additionally, this work analyzed the energy of the production and operation stages of bionanocompounds. Moreover, our suggested LCA highlighted the steps in the synthesis of bionanocompounds that have the most negative effects on the environment and human health.

An initial stage that involved the fabrication of the bionanocompounds was considered. This stage was analyzed by applying the LCA methodology, and the results showed that bionanocompounds had almost 95% more impacts than water. This result was attributed to the significant amount of energy and raw materials required to produce the bionanocompounds. Therefore, the latter showed a lower environmental performance in all impact categories when compared with water. 

A second stage involved the reusage of each bionanocompound during their operation in four work cycles. The LCA results related to this stage revealed that bionanocompounds had a 91% lower impact across all impact categories. These findings can be explained by the high defrosting time and the omission of energy and raw materials used during the manufacturing stage. Moreover, the results suggested an estimated total energy saving of 47% by the magnetite + INA bionanocompound and 7% by the silica + INA bionanocompound when compared with water. Therefore, our results indicated that bionanocompounds are a promising alternative for freezing applications, considering the minimal implications on the environment and human. Additionally, future work can address the large-scale production of bionanocompounds, including new eco-friendly alternatives in the synthesis process. Furthermore, a complete LCA study can be performed by analyzing the scaled-up production, usage, and final disposition of the bionanocompounds in freezing applications.

## Figures and Tables

**Table 1:** Inventory report for each stage considered.

Stage	Process	Inventory	Amount	Unit
Manufacturing	Raw materials	Magnetite nanoparticles (Fe_3_O_4_)	0.1	g
		Silica nanoparticles	0.1	g
		Energy (precision scale)	0.0033	kWh
		Water consumption	0.005	L
	Re-suspension	Tetramethylammonium hydroxide (TMAH)	0.02	L
		Energy (ultrasonic bath)	0.08	kWh
		Water consumption	0.1	L
	Silanization	(3-Aminopropyl) triethoxysilane (APTES)	0.001	L
		Energy (ultrasonic bath)	0.04	kWh
		Water consumption	0.1	L
	Crosslinker	Glutaraldehyde (GLU)	0.002	L
		Energy (vortex)	0.0006	kWh
		Water consumption	0.1	L
		Energy (refrigerator)	0.144	kWh
	Second layer	INA protein (Snowmax)	0.01	g
		Energy (vortex)	0.0006	kWh
		Water consumption	0.001	L
		Energy (refrigerator)	3.456	kWh
	Washing and re-dispersion	Energy (ultrasonic bath)	0.3	kWh
		Energy (centrifuge)	3.5	kWh
		Energy (vortex)	0.044	kWh
		Water consumption	0.15	L
Operation	Operation	Magnetite + INA bionanocompound	40	mL
		Energy (refrigerator)	3.943	kWh
		Silica + INA bionanocompund	40	mL
		Energy (refrigerator)	7.487	kWh
		Water consumption	40	mL
		Energy (freezer)	0.267	kWh

**Table 2:** Total impact assessment results for bionanocompounds and water, including the manufacturing and operation stages.

Impact Categories	Unit	Magnetite + INA	Silica + INA	Water
Human toxicity, non-carcinogenic effects	CTU	5.86 × 10^−5^	1.04 × 10^−4^	1.12 × 10^−4^
Human toxicity, carcinogenic effects	CTU	7.79 × 10^−9^	1.38 × 10^−8^	1.49 × 10^−8^
Ecotoxicity of freshwater	CTU	0.55	0.98	1.06
Climate change	kg CO_2_-Eq	1.45	2.57	2.77
Resource depletion, minerals and metals	kg Sb-Eq	1.86 × 10^−6^	3.35 × 10^−6^	3.55 × 10^−6^
Resource depletion, dissipated water	m^3^ water-Eq	0.70	1.23	1.33
Photochemical ozone formation	kg NMVOC-Eq	4.34 × 10^−3^	7.69 × 10^−3^	8.29 × 10^−3^
Freshwater and terrestrial acidification	mol H^+^-Eq	1.13 × 10^−2^	2.00 × 10^−2^	2.15 × 10^−2^

## Data Availability

The data and contributions presented in the study are included in the article. Further inquiries can be directed to the corresponding author.

## Supplementary Materials

The following supporting information can be downloaded at https://www.mdpi.com/article/10.3390/polym15061457/s1. Figure S1: Results of the impact assessment results for the manufacturing and operation stages: (a) non-carcinogenic human toxicity effects, (b) carcinogenic human toxicity effects, (c) ecotoxicity of freshwater, (d) climate change, (e) resource depletion of minerals and metals, (f) resource depletion of dissipated water, (g) photochemical ozone formation, and (h) freshwater and terrestrial acidification. Figure S2: Analysis of the contribution of the magnetite + INA bionanocompound, the silica + INA bionanocompound, and water: (a) manufacturing stage and (b) operation stage.
