# Supplementary material for: Life Cycle Assessment of Functionalized Bionanocompounds with Ice Nucleation Protein for Freezing Applications
Source: Polymers (Basel). 2023 Mar 15;15(6):1457. doi: 10.3390/polym15061457 (PMC10058881; doi:10.3390/polym15061457)
Supplement: Supplementary file 1 [file polymers-15-01457-s001.zip › polymers-2036856-supplementary.pdf]

polymers

Supplementary material

Life Cycle Assessment of Functionalized Bionanocompounds with Ice Nucleation Protein for Freezing Applications

Olga P. Fuentes [1] and Johann F. Osma [1,2,*]

[1] Department of Electrical and Electronic Engineering, Universidad de los Andes, Cra. 1E No. 19a-40, Bogota 111711, Colombia
[2] Department of Biomedical Engineering, Universidad de los Andes, Cra. 1E No. 19a-40, Bogota 111711, Colombia
* Correspondence: jf.osma43@uniandes.edu.co; Tel.: +57-6013394949

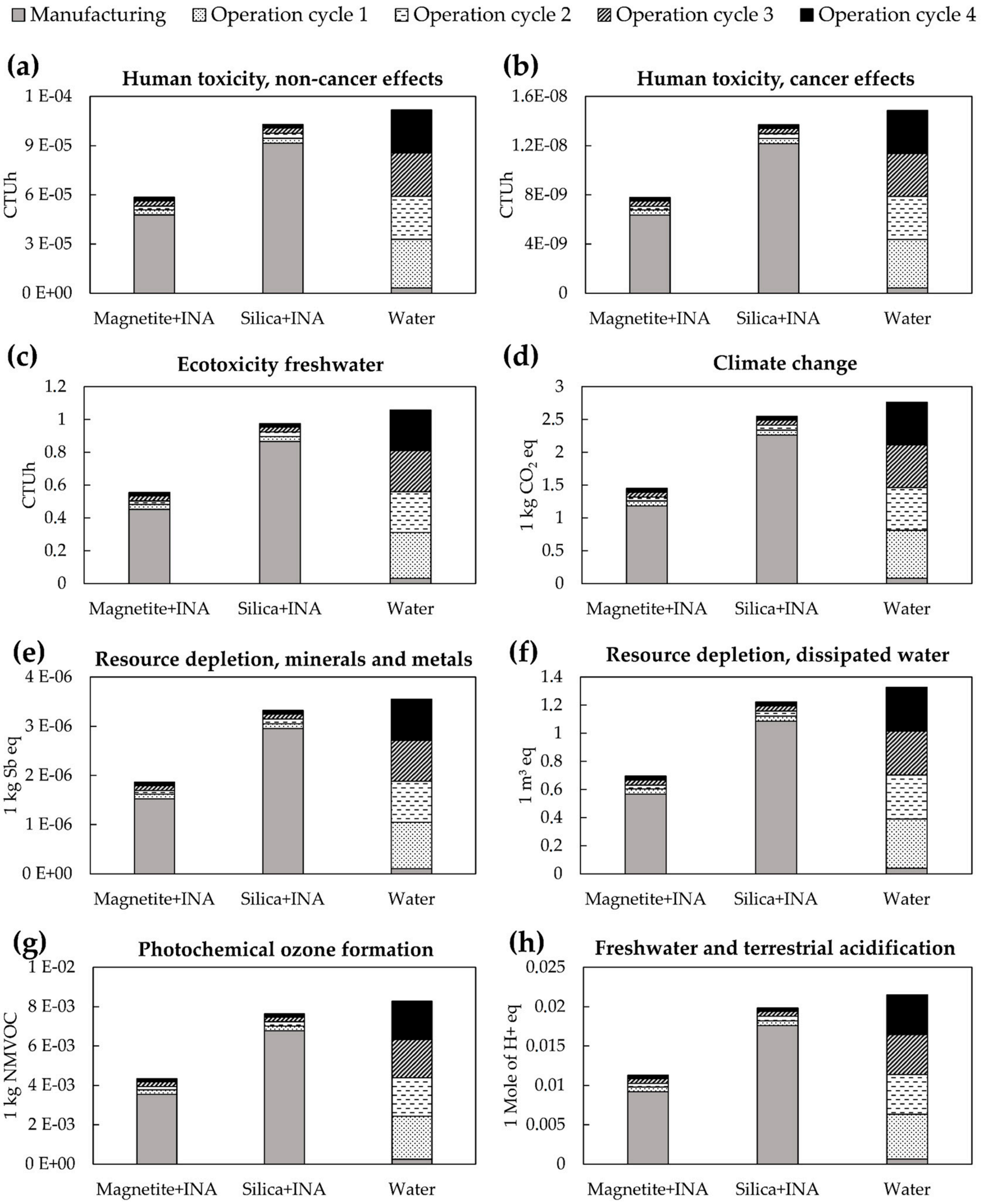

Figure S1. Impact assessment results of the manufacturing and operation stages: (a) Human toxicity, non-cancer effects, (b) Human toxicity, cancer effects, (c) Ecotoxicity freshwater, (d) Climate change, (e) Resource depletion, minerals and metals, (f) Resource depletion, dissipated water, (g) Photochemical ozone formation, (h) Freshwater and terrestrial acidification.

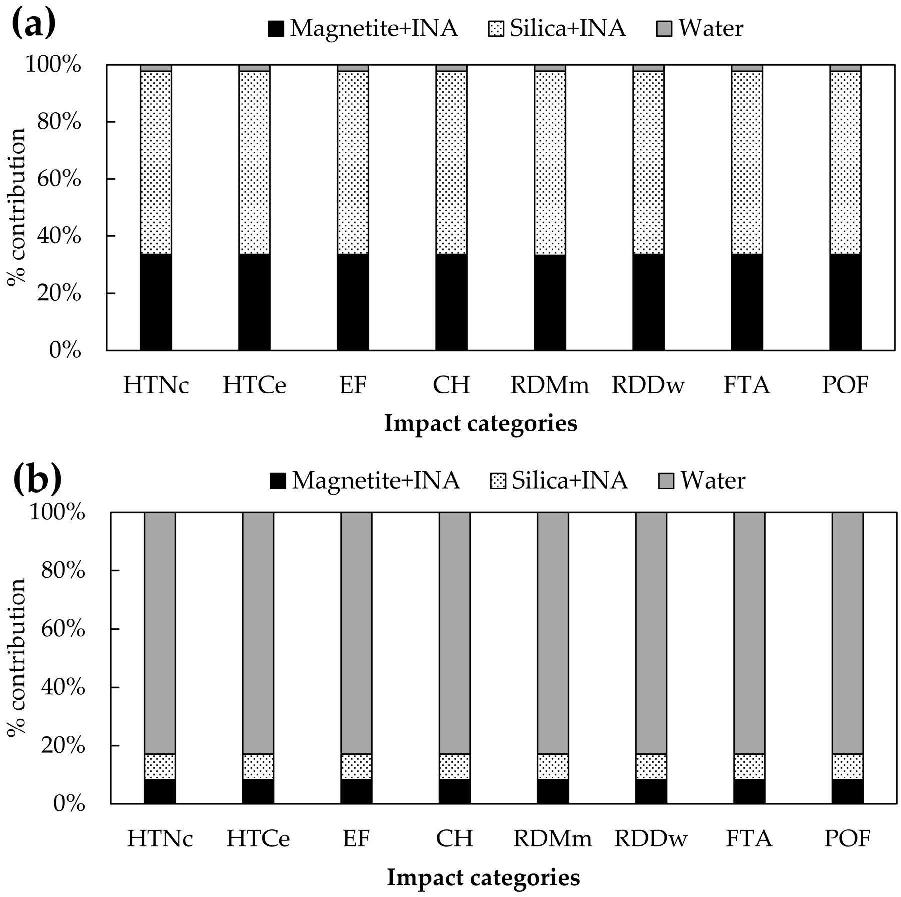

Figure S2. Contribution analysis of Magnetite+INA bionanocompound, Silica+INA bionanocompound, and water: (a) Manufacturing stage and (b) Operation stage.
